# Application of Atomic Dielectric Resonance Spectroscopy for the screening of blood samples from patients with clinical variant and sporadic CJD

**DOI:** 10.1186/1479-5876-5-41

**Published:** 2007-08-30

**Authors:** Timothy J Fagge, G Robin Barclay, G Colin Stove, Gordon Stove, Michael J Robinson, Mark W Head, James W Ironside, Marc L Turner

**Affiliations:** 1 National CJD Surveillance Unit & Division of Pathology, University of Edinburgh School of Molecular and Clinical Medicine, Western General Hospital, Edinburgh EH4 2XU, UK; 2 SNBTS Adult Cell Therapy Group, Scottish Centre for Regenerative Medicine, University of Edinburgh School of Clinical Sciences, The Chancellor's Building, 49 Little France Crescent, Edinburgh EH16 4SB, UK; 3 ADROK Ltd (formerly Radar World Ltd), Waterloo House, 17 Waterloo Place, Edinburgh, EH1 3BG, UK

## Abstract

**Background:**

Sub-clinical variant Creutzfeldt-Jakob disease (vCJD) infection and reports of vCJD transmission through blood transfusion emphasise the need for blood screening assays to ensure the safety of blood and transplanted tissues. Most assays aim to detect abnormal prion protein (PrP^Sc^), although achieving required sensitivity is a challenge.

**Methods:**

We have used innovative Atomic Dielectric Resonance Spectroscopy (ADRS), which determines dielectric properties of materials which are established by reflectivity and penetration of radio/micro waves, to analyse blood samples from patients and controls to identify characteristic ADR signatures unique to blood from vCJD and to sCJD patients. Initial sets of blood samples from vCJD, sCJD, non-CJD neurological diseases and normal healthy adults (blood donors) were screened as training samples to determine group-specific ADR characteristics, and provided a basis for classification of blinded sets of samples.

**Results:**

Blood sample groups from vCJD, sCJD, non-CJD neurological diseases and normal healthy adults (blood donors) screened by ADRS were classified with 100% specificity and sensitivity, discriminating these by a co-variance expert analysis system.

**Conclusion:**

ADRS appears capable of recognising and discriminating serum samples from vCJD, sCJD, non-CJD neurological diseases, and normal healthy adults, and might be developed to provide a system for primary screening or confirmatory assay complementary to other screening systems.

## Background

The human prion diseases or transmissible spongiform encephalopathies (TSEs) are a group of fatal neurodegenerative disorders believed to be caused by a post-translational conformational change in cellular prion protein from its soluble form (PrP^C^) to a pathogenic protease resistant isoform PrP^Sc ^[1]. The most common of these is sporadic Creutzfeldt-Jakob disease (sCJD) but a variant form of CJD (vCJD) was identified in the UK in 1996 [2] and has been linked to human infection by the bovine spongiform encephalopathy (BSE) agent. Existing clinical tests for Creutzfeldt-Jakob disease (CJD) can establish a diagnosis of probable variant or probable sporadic CJD during the clinical phase of disease, but a definitive diagnosis depends on post-mortem examination of the brain. Moreover there is currently no practical way of determining whether an individual who is not manifesting symptoms is incubating the disease.

The presence of PrP^Sc ^in the peripheral tissues of patients with vCJD [3,4] and experimental transmissions of BSE and natural scrapie between sheep by blood transfusion [5,6] raised the possibility that iatrogenic transmission in humans by blood transfusion could occur. This risk has unfortunately been confirmed by recent reports of transmission of vCJD by blood transfusion [7-10]. The annual number of deaths from variant CJD has been declining since a first peak in 2000. However, the prevalence of infected individuals in the general UK population, as judged by a retrospective tonsil and appendix tissue study, appears to be higher than would be expected from the mortality rates, pointing to substantial numbers of sub-clinically infected individuals in the general population [11,12]. To date all reported clinical cases of vCJD have been homozygous for methionine at codon 129 of the prion protein gene (PRNP), and there has been uncertainty over the susceptibility of the heterozygous and valine homozygous genetic sub-groups. There are now good reasons to believe that all PRNP codon 129 genotypic groups are susceptible to infection [9,13] and it is possible that heterozygous and valine homozygous individuals may exhibit prolonged incubation periods or might remain in a sub-clinical state throughout life. In the absence of a screening test for vCJD these individuals could be a major source of iatrogenic transmission. Current methods that might identify these pre- or sub-clinical individuals, such as lymphoreticular biopsy, are highly invasive and clearly impractical as screening measures for blood donations. A screening assay that could be applied to a routine blood sample is urgently required.

Although there is a presumption that PrP^Sc ^is the infectious agent, the association of infectivity with blood is poorly understood. There is growing evidence from studies in mice and hamsters that initial TSE replication occurs in lymphoreticular tissues [14,15] prior to invasion of the central nervous system (CNS) via the sympathetic nervous system [16-19]. Recent reports of the transmission of natural scrapie and experimental BSE between sheep by whole blood and buffy coat transfusion support the theory that infectivity is associated with, but not restricted to, the white cell component [20]. Given that levels of detectable PrP^Sc ^and infectivity in peripheral lymphoreticular tissues such as spleen and tonsil in patients with vCJD are 2–3 logs lower than levels detected in the CNS [4,21], it is likely that PrP^Sc ^is present at very low concentrations in peripheral blood. Attempts to detect PrP^Sc ^in human buffy coat by Western blot have thus far proven unsuccessful [4]. Intracerebral inoculation of human buffy coat from clinical vCJD cases into susceptible mouse models has also failed to demonstrate infectivity [22], although this could be a reflection of species barrier and/or the small numbers of animals used in addition to the limited volume of inoculum that can be delivered intracerebrally. Because of its evident very low level, pre-mortem tests designed for the detection of any PrP^Sc ^present in blood/body fluids would require a high level of sensitivity, probably several logs greater than those diagnostic tests already approved by the European Commission and in place for post mortem TSE disease confirmation in slaughterhouse cattle and sheep. Many of these tests rely upon proteolytic treatments to digest PrP^C ^and may lack the sensitivity required for blood screening as they may remove protease sensitive isoforms of disease associated prion protein. There are a number of emerging diagnostic tests in development for pre-mortem diagnosis which may possibly be capable of detecting PrP^Sc ^in peripheral blood, amongst which the protein misfolding cyclic amplification (PMCA) appears particularly promising [23,24], but none of these has yet been validated in extensive study of human blood samples. These have recently been reviewed by Brown [25].

The difficulties of achieving the sensitivity needed for the detection of PrP^Sc ^has shifted efforts to an alternative approach of screening for biomarkers other than prion protein. Potential candidates assays have been recently reviewed by Parveen et al. [26]. However most lack the specificity and sensitivity to be useful as diagnostic screening tests and would need to be employed with an array of confirmatory assays. At present many do not have data on studies of CJD in humans. Despite this there exist a small number of promising alternate approaches. Amongst these, emerging spectroscopic methods, for example the use of Fourier transform infrared spectroscopy (FT-IR) as a test for BSE in cattle or preclinical scrapie in hamsters, have been described [27,28]. Analysis of blood serum by FT-IR supported by artificial neural network or multivariable pattern recognition analysis allowed the sensitive and specific discrimination between healthy and infected animals [28,29]. In this paper we report on the development and assessment of atomic dielectric resonance (ADR) spectroscopic techniques in an analysis of clinical blood samples from patients with CJD. This also employs Fourier transformation and complex pattern analysis.

ADR is a new technology exploiting novel properties of resonance of radio waves from X-band and C-band radar developed for aerospace and ground remote sensing, imaging, and materials detection and recognition through surface boundaries. This technology has been recently described in a patent [30]. ADR spectroscopy (ADRS) exploits dielectric properties of materials which determine reflectivity and penetration of radio/micro waves, allowing the use of electromagnetic energy as a means of remotely detecting and measuring target characteristics. Different materials have unique energy absorption and reflection properties and provide spectra in the broad range of frequencies employed that provide signatures for materials recognition and matching (typecasting). ADRS has been developed for a broad range of applications predominantly in geophysical, engineering or security fields which have not been published because of commercial or other sensitivities. Several small scale biological laboratory or clinical (patient or tissue) studies have indicated potential utility of ADR in biomedical investigation both in subsurface imaging and in materials characterisation, and the apparatus has been approved as a medical device for use in human subject studies as well as in the laboratory. However, interest in application of ADR in this area is novel and as yet no formal studies in a biomedical context have been published. ADR employs very low energy emission, and is a reagent free non-destructive non-consuming test. Unlike infrared spectroscopy, there is no requirement for drying or other special treatment of test materials. Preliminary (unpublished) studies by us of histology slides (remaining from a separate histology study) of brain sections from experimental scrapie infected mice and uninfected controls illustrated that it was possible to clearly distinguish scrapie infected mice at a range of stages of scrapie infection from normal controls by match ranking of ADR spectra taken from these slides [31]. Results are presented here which demonstrate how characteristic ADR signatures can be exploited to discriminate between the blood of patients with clinical sCJD, those with clinical vCJD, those from non-CJD neurological control patients and those from healthy adult (blood donor) controls.

## Methods

### Collection of blood samples

Anonymised whole-blood samples from healthy adult donors were collected by the Scottish National Blood Transfusion Service Edinburgh and stored for 24 hours at 4°C to mimic the conditions of collection of samples from CJD patients transported to the National CJD Surveillance Unit in Edinburgh from around the UK. Blood from CJD patients and neurological controls were obtained by the National CJD Surveillance Unit primarily for genetic analysis: however consent was obtained for research use of any remaining specimen. Whole blood samples from vCJD, sCJD patients, and neurological controls were used in ADRS studies. Informed consent was also obtained from donors for experimentation. All vCJD and sCJD cases had a probable or definitive diagnosis based on internationally established criteria [32,33]. The neurological control group were samples from patients referred to the CJD Surveillance Unit who subsequently did not meet criteria for a diagnosis of definite or probable CJD and who can be assumed to be suffering from neurological diseases other than CJD or other prion disorders. Whole blood samples were handled, separated, and stored in an identical manner to ensure groups were directly comparable in scientific investigations. Samples were collected into 9 mL vacuettes containing 1 mL of 3.2% tri-sodium citrate (Greiner Bio-One, Ltd, Gloucestershire, UK).

### ADRS apparatus and sampling

The ADRS hardware for this study consisted of a radiowave sampling control unit (RCU), a pulse generator (PG) and a Test Sample Chamber (TSC) in which individual samples were placed which incorporated a transmitting and receiving antenna array. Resonant scan-returns were received from the illuminated samples in their containers in a 90 degree cross polarised mode and then transferred directly to a PC where the image data were digitally stored.

Samples (500 μL) were dispensed into the same batch of 2 mL polypropylene tubes with caps and placed onto a spot within the sample chamber. Four scans of each sample were taken. Scans were also taken on empty tubes for subtraction of spectra. As far as we could ensure, no artefacts were introduced from any differences in handling, storage, or dispensing of samples. Sample codes for each sample were recorded with spectral data and stored in the linked data acquisition computer.

Samples were pulsed with radar in the spectral range 0–25000 MHz and spectral frequency and energy measurements (images) were classified using energy bins. The image data were first subjected to fast Fourier transform (FFT) analysis using the RADAMATIC software, proprietary software developed by Radar World Ltd, which is optimised for analysing the Atomic Dielectric Resonance behaviour of materials when subjected to a coherent beam of lased invisible light photons. The bandwidth of the pulsed transmit (Tx) energy was 1 GHz but the ADR spectral responses were analysed from the received (Rx) digital signals by FFT methods using 1024 point samples in each case from 100 MHz to 51.2 GHz.

### First Study design

Whole blood samples from 5 different patients or blood donors for each of vCJD, sCJD, neurological controls and healthy adult blood donor control groups were assembled for testing in unlabelled 2 ml polypropylene tubes. Of these 2/5 were left un-blinded and used as controls for training, leaving the remaining 3/5 samples as blinded test samples which were randomised. Clinical and control sample groups were assigned alphabetic codes to ease data analysis, vCJD: A; sCJD: B; Neurological controls: C; Healthy blood donor controls: D, E.

### Primary analysis of ADR spectra

The FFT generated spectral data sets for clinical and control samples were analysed using RADAMATIC ADR and Image Analysis software developed and owned by Radar World Ltd. In classic EM Theory, the EM properties of the mediums of propagation (air, water, soil, rock and biological materials, for example) there are three key variables which are usually studied:

(1) Dielectric permittivity (ε)

(2) Magnetic permeability (μ), and

(3) Electric conductivity (σ)

The mathematical and statistical methods of ADR analysis produce synthetic relationships which can then be used to compute very precise values of ε, μ and σ. The software analysis incorporated is primarily concerned with identifying materials which have to be mapped or assessed in some way. If change detection is important, then the software is concerned with mapping the extent or spread of the material in question over specific time scales to monitor increased or decreased spatial or volume extents. In order to unambiguously identify the material in question the software does not necessarily need to know the specific values of ε, μ and σ, which, *per se*, may not necessarily identify the unknown material in question, but it may be necessary to establish 10 or 12 other mathematical or statistical relationships related to the energy-frequency spectra of the unknown material in question, to establish from the ADR control database of such parameters unquestionably what the material's code is by the software's logical expert systems method of discrimination.

Primary data analysis consisted of the subtraction of ADR image data generated by scanning the empty tube from ADR spectral image data generated by each test sample. The resultant data represented true ADR image data for blood samples alone, with no contribution from the sample tube. These data were then re-compiled into an ADR spectral data base where rank matching of the 10 or 12 established spectral relationships were tested for within group and between group relationships. An expert system was developed using the control logic results of the analysis of variance tables and this finally was able to identify the unique code attributed to each sample tested. The final results were proved to be 100% correct on two separate occasions when double blind test procedures were adopted. It is important to add that 100% accuracy is only achieved in identifying new materials by this method provided that a set of at least 20% control samples are "trained" by this ADR spectroscopic method and the logic control relationships are mathematically defined from this result for the Expert Systems Analysis Method which is used to identify the unknown (blinded) samples.

### Match Ranking Analysis

In an attempt to identify blinded samples, unprocessed Fourier transformed ADR spectra were match ranked. This method employed a single scan of a known training sample used as a reference spectrum against which the remaining scans of control and test samples were matched. The closeness of the matching was based upon determining the correlation coefficient values and setting them in order, therefore the closer to 1 or in this case 100% the more exact the match. It was considered that this approach may allow classification of blinded samples by measuring their level of correlation to a known reference sample.

### Analysis of variance classification: Expert systems analysis

An expert system was developed based upon analysis of variance of frequency data within groups and between groups. Studying firstly the similarities, and then the differences between different samples groups produced a series of relationships which were used to develop logic relationships and an expert system. The identification of robust sets of P-value relationships for each sample group allowed the classification of test samples. The P-ratios of variance between and within groups were calculated by rank and cluster analysis of ADR-ratio data using Radamatic software.

### Second Study design

To validate the ability of expert systems analysis methods to correctly identify and distinguish between clinical CJD and control samples a second study was undertaken. 10 whole blood samples were obtained from the National CJD Surveillance Unit frozen archives. All samples were blinded and included 4 vCJD, 3 sCJD, and 3 samples from non-CJD neurological controls. It was ensured that none of the samples had been included in the first ADR spectroscopy study. Thawed whole blood samples (0.5 ml) in 2 ml storage tubes were scanned 4 times and scans of an empty storage tube were subtracted from sample scans so that spectral data represented the blood sample alone. Spectral data from this study was classified using the data obtained in the first study.

## Results

### First study

In the first study ADRS spectra from 4 scans each were obtained from whole blood samples from clinical patients with sCJD and vCJD and healthy adult donors and neurological control patients. The first study was essentially used for system evaluation and training purposes, to an extent to determine how much training of the system on known samples was required for unambiguous discrimination of the remaining samples, and which analysis procedures could achieve this. Spectra were analysed by combinations of analysis techniques to assess the best classification methods. For primary analysis spectral data was compiled into tables which show ADR parameters (F1, F2) between 1–99% energy bins and the ADR ratio which is a measure of the rate of change of resonance with frequency cut-off per energy bin. There are a number of important observations which can be made from studying the data presented in Table 1 which summarizes the first 20% E-bin results and the last 10% E-bin results for individual samples from the vCJD and the sCJD sample groups from the detailed fast Fourier transform (FFT) tables computed by the Radamatic software. At the 99% energy level the frequency resonance is 947 MHz for vCJD compared with 1320 MHz for sCJD. However the actual resonance value at this level as computed by the standard variance f2 ADR parameter statistic is much higher (+/-32.3 MHz) for vCJD than that (+/-13.6 MHz) for sCJD. This is a significant result, which can be summarized in the ADR-Ratio. This is an inverse index and the lower the ADR-Ratio the higher the ADR parameter. In this case vCJD yields an ADR-ratio of 29.348 at the 99% energy-bin compared with 97.216 for sCJD. There is also a distinct trend of resonance variability with vCJD compared with sCJD. For the 1st 7 energy-bins the ADR-ratio for sCJD is constant at 15.7, whereas the ADR-ratios for vCJD vary considerably from 4 (1%), 11 (2%), 31 (3%), 49 (4%), 32 (5%), 21 (6%) and 15 (7%).

**Table 1:** ADR parameters for individual vCJD and sCJD samples from the first study

***E(%)***	***f1(MHz)***		***f2(MHz)***		***ADR-Ratio***	
	vCJD	**sCJD**	vCJD	**sCJD**	vCJD	**sCJD**
1	186.0	**22.0**	42.0	**1.4**	4.412	**15.700**
2	212.0	**45.0**	17.8	**2.9**	11.939	**15.700**
3	230.0	**67.0**	7.4	**4.3**	31.063	**15.700**
4	248.0	**90.0**	5.0	**5.7**	49.842	**15.700**
5	266.0	**112.0**	8.1	**7.1**	32.733	**15.700**
6	284.0	**134.0**	13.4	**8.6**	21.246	**15.700**
7	302.0	**157.0**	19.1	**10.0**	15.858	**15.700**
8	321.0	**179.0**	24.2	**11.0**	13.264	**16.292**
9	339.0	**199.0**	25.6	**10.2**	13.229	**19.478**
10	357.0	**214.0**	24.0	**10.9**	14.851	**19.674**
11	375.0	**229.0**	20.4	**11.0**	18.373	**18.048**
12	391.0	**245.0**	15.6	**14.7**	25.022	**16.605**
13	394.0	**260.0**	10.9	**16.9**	36.211	**15.393**
14	397.0	**275.0**	7.4	**19.1**	53.853	**14.390**
15	400.0	**290.0**	6.0	**21.4**	66.388	**13.559**
16	403.0	**305.0**	6.1	**23.7**	66.204	**12.866**
17	406.0	**320.0**	6.1	**25.9**	66.019	**12.351**
18	409.0	**335.0**	6.2	**26.7**	65.832	**12.516**
19	412.0	**350.0**	6.3	**27.1**	65.644	**12.887**
20	415.0	**365.0**	6.3	**27.2**	65.456	**13.394**
						
90	717.0	**904.0**	22.5	**6.1**	31.810	**147.013**
91	727.0	**915.0**	21.8	**5.6**	33.446	**162.748**
92	738.0	**927.0**	22.3	**5.1**	33.075	**181.498**
93	748.0	**938.0**	24.1	**4.6**	31.050	**204.118**
94	759.0	**950.0**	28.2	**4.1**	26.890	**231.745**
95	769.0	**961.0**	34.6	**3.6**	22.233	**265.856**
96	780.0	**973.0**	42.9	**5.6**	18.170	**174.787**
97	832.0	**54.0**	51.2	**26.7**	16.253	**39.452**
98	889.0	**173.0**	48.0	**22.1**	18.539	**53.040**
99	947.0	**1320.0**	32.3	**13.6**	29.348	**97.216**

Data shown are the ADR parameters f1 and f2 and the ADR-ratio for individual vCJD and sCJD samples for energy levels E-bin 1% to E-bin 20%, and E-bin 90% to E-bin 99%.

**Figure 1:**
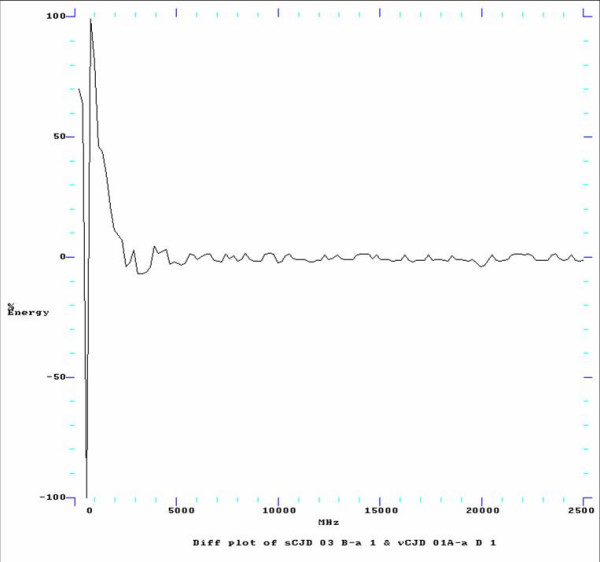
**FFT Difference plot comparing vCJD spectra with sCJD spectra**. Illustrates the FFT Difference plot by subtracting an individual vCJD spectra from an individual sCJD spectra. This shows the variability of vCJD over sCJD, particularly from 3000 MHz to 25000 MHz and is again a useful diagnostic indicator of differences.

To highlight spectral differences between samples, the amplitude (or maximum wave displacement of absorption spectra) of the same vCJD and sCJD samples were plotted against energy in decibels (Db). Figure 1 shows the FFT difference plot created by subtracting the vCJD spectral data from the sCJD spectral data. This illustrates the variability of vCJD over sCJD, particularly from 3000 MHz to 25000 MHz and could be a useful diagnostic indicator of differences between groups. The spectral detail represents all molecular groups contained in the whole blood sample, and it is not possible at this time to assign spectral signals to specific molecular constituents of the blood. It is not apparent at this stage of the analysis whether these interesting spectral differences between individual samples are associated with the clinical class of the samples. They may merely reflect differences such as blood type between individuals and factors which are not associated with disease status. A detailed analysis of much larger sample numbers from each clinical class may permit the identification of particular spectral patterns able to rapidly distinguish vCJD from sCJD or from healthy adult blood samples, without the need for complex analysis.

Relatively simple match ranking data analysis techniques, based on correlation, were used first to analyze ADR spectra to identify unknown samples by studying the correlation coefficient values when matched against the reference spectrum of a single known training sample. There was discrete clustering of a large proportion of samples according to class, which indicated strong clinical features of many samples which dominated over the individuality of control samples. Clinical and control samples segregated at opposite ends of the range which infers the presence of a unique combination of physiological factors which distinguish them. However, segregation of clinical and control groups was not absolutely discrete and did not improve the ability to identify unknown samples with confidence. Additional strategies for primary analysis including filtering spectral data and image analysis were tried to identify blinded samples, but it became evident that match ranking was not sufficiently reliable to detect the identity of all samples. At this stage the identities of all clinical and control samples in the first study were un-blinded to allow the entire data set to be used to develop expert system analysis algorithms for identification of the classes of clinical and control whole blood samples.

To develop the expert system the 4 scans each of the first training sample from each clinical and control sample class were used to compute P-value tables, producing a series of 13 parameters to define each sample group. Each parameter produced a series of defining statistics and relationships between sample classes, the summation of which led to the development of an expert system of analysis able to characterise the different sample classes. A P-value computation to produce revised parameters enabled the correct classification of all previously blinded samples with 100% specificity and accuracy. Due to sample variability between patients the identification of the class of 20% of all samples was necessary for adequate training to ensure correct classification of the remaining 80% of samples at the 0.001% significance level or 0.05% confidence level.

### Second study

To validate the P-value co-variance expert system a small second blinded study of clinical and neurological control whole blood samples was undertaken. Spectral data was handled as for the first study. Frequency analysis of ADR spectra produced ADR-ratio data, and rank and cluster analysis using Radamatic software was used to produce P-ratios of variance which produced a range of indices used as discreet classifiers for the unknown samples. The P-variance data classification parameters defined by training the system with samples in the first study were used to correctly identify the blinded samples in this subsequent study by studying the percentage correlation of oscillation between the unknown sample and the classification parameters which define each clinical and control group. These unknown samples were identified with 100% specificity and accuracy.

## Discussion

We have presented proof of principle of a potential method employing ADR spectroscopy and an expert systems statistical spectral analysis for the classification of sCJD and vCJD clinical and neurological control and healthy adult whole blood samples. This classification system was developed in an initial study and validated by a subsequent small blind study where samples were correctly identified with 100% sensitivity and specificity. The findings suggest that there are distinct physiological markers detectable in clinical whole blood samples from vCJD and sCJD patients by ADR spectroscopy. These methods present a rapid, non-destructive reagent-free test system for the clinical diagnosis of patients with CJD that is not (knowingly) dependent on the detection of PrP^Sc ^for their classification. The number of samples analysed in these studies is small. Nevertheless the probability that these could be discriminated with 100% specificity and sensitivity by chance is extremely low. A much larger validation study of healthy adult controls, clinical CJD, non-CJD control samples, along with samples from patients with a wide spectrum of neurological, infectious and non-infectious conditions is required to assess to what extent the expert system statistical parameters are specific for each group or whether the spectral structural compositional alterations characteristic in CJD blood samples are shared by other neurological and infectious diseases.

ADR spectral analysis reveals that there are distinct structural compositional features being detected in clinical CJD blood samples. As for FT-IR studies which have discriminated TSE infection in sera from different species [34], the exact nature of the ADR spectral alterations is unknown. They are more likely to be due to distinct combinations of blood physiological features for particular disease classes rather than due to the detection of low levels of PrP^Sc^. Detection of PrP^Sc ^was the assumption in a preliminary (unpublished) mouse scrapie study where PrP^Sc ^in brain tissue is prevalent, and where correlation-based match ranking was sufficient to discriminate between normal mouse brain (which included some cases of non-scrapie pathological abnormalities), and mouse brain from a range of stages of scrapie infection [31]. ADRS is a new technology and while it is excellent at recognising materials by comparison to databases it can not yet assign spectral features to specific molecular entities as may be possible for some interpretations of FT-IR [34]. The advantage that ADRS may have over FT-IR even at this early stage of development is that there are no special requirements for sample preparation such as drying, and that a very wide spectral frequency range is used which provides more possibilities for discriminatory analysis.

Indirect improvements in sensitivity may be possible. For example, the system might be trained to recognise and cancel out intrinsic differences associated with different blood types in test samples, so as to increase the reliability of the system analysis for CJD diagnosis. It may also be possible to simplify methods by separating blood into constituent components and determining whether some component is more strongly associated with the ability to be discriminated by ADRS. For example, CJD plasma samples might be more easily identified if the spectral noise of blood samples is reduced by removing cells and cell debris if these do not contribute to the spectral discrimination. Investigations of pre-clinical blood samples from experimental TSE animal models and from individuals known to have received infected blood, if available, would also be important in the development of a pre-mortem screening test using ADR spectroscopy. At this stage, the analysis of data is greatly dependent on the expertise and intuition of the developers of ADRS at Radar World Ltd. However, when the analysis parameters are fully validated and assessed in extended studies, and if necessary improved, the analytical algorithms can be incorporated into hardware or software which can be employed by inexperienced users in specific applications.

The ADRS method described here presents a unique approach for the diagnosis of CJD using blood, and it is possible that with further study these methods may be adapted for the screening of donated blood for vCJD in the pre- or sub-clinical phase to ensure the removal of infected blood components from the supply chain and consequently a reduction in the risk of iatrogenic transmission of vCJD. In effect, this could restore access to blood donor populations currently proscribed in the UK, such as those who have previously received blood transfusion or blood products, and restore access to UK blood donor plasma for its fractionation into blood products. If other tests suitable for screening blood samples for CJD are developed, they are likely to be based on different technologies. An ADRS-based test would represent a complementary partner for such a test in screening systems where one could be employed as the primary screening system and the other as a validation system. Speculatively, it is also possible that since ADRS is very low energy and suitable for use as a medical device, and conformations of ADRS hardware can be developed for non-invasive patient scanning to produce images and spectra from tissue within the patient, ADRS could be developed and systems trained to identify tissues affected by TSE disease. Such a development might be capable of detecting deposition of PrP^Sc ^in lymphoid and other tissue in vCJD infection, since we know from preliminary studies in scrapie infected mouse brain that PrP^Sc ^has a strong dielectric signal in ADRS, and which would also be a valuable contribution to identifying subclinical vCJD infection.

## Conclusion

We have established in a proof of concept study that ADRS can discriminate between blood samples from patients with clinical vCJD, sCJD, non-CJD neurological diseases and from normal healthy adults with 100% sensitivity and specificity. A larger validation is now required to assess the specificity of the expert system statistical parameters for CJD blood samples compared to a wide range of other neurological and infectious diseases. Further studies are required to investigate whether ADRS could be used to recognise pre- or sub-clinical vCJD cases, with a view to determining whether ADRS could be developed as a blood screening system.

## Competing interests

The authors declare that they have no competing interests. Rader World Ltd participated on a voluntary basis out of scientific curiosity and is not currently developing a commercial interest in this field.

## Authors' contributions

GRB and GCS conceived of the study, and participated in its design with TJF and MWH. JWI and MLT authorized access to patient and blood donor samples for the study and contributed clinical expertise and oversight to the study design and critical comment on the results. TJF prepared all samples and participated in their screening with GCS and MJR. Data was analysed by GCS, MJR and GS in liaison with TJF and GRB. TJF and GRB drafted the manuscript and all authors read and approved the final manuscript.
